# First *mec*C and *mec*A Positive Livestock-Associated Methicillin Resistant *Staphylococcus aureus* (*mec*C MRSA/LA-MRSA) from Dairy Cattle in Malaysia

**DOI:** 10.3390/microorganisms8020147

**Published:** 2020-01-21

**Authors:** Erkihun Aklilu, Hui Ying Chia

**Affiliations:** Faculty of Veterinary Medicine, University Malaysia Kelantan, Locked Bag36, Pengkalan Chepa 16100, Kota Bharu, Kelantan, Malaysia; chia.hui.ying@hotmail.com

**Keywords:** antimicrobial resistance, MRSA, *mec*C, LA-MRSA, *S. aureus*, dairy cattle, zoonotic risks

## Abstract

Livestock associated Methicillin resistant *Staphylococcus aureus* (*S. aureus*) (LA-MRSA) was reported to be zoonotic and may transmit to farmers and veterinarians. The objectives of this study were to investigate the occurrence of LA-MRSA from dairy cattle and to evaluate the antimicrobial resistance profiles of the isolates. A total of 63 milk and 32 nasal swab samples were randomly collected from dairy cattle. The samples were processed to isolate *S. aureus,* MRSA and LA-MRSA using both phenotypic and molecular methods using PCR. The confirmed *S. aureus* isolates were cultured on oxacillin resistant screening agar base (ORSAB) to detect MRSA and the isolates were further confirmed by PCR targeting the *mec*A gene. Detection of the novel *mec*A gene, *mec*C gene was conducted by PCR amplification. The antimicrobial susceptibility tests were conducted using disc diffusion method. Results revealed 17/95 (17.89%) and 15/95 (15.79%) were positive for *mec*A and *mec*C genes respectively. Out of the 15 *mec*C positive isolates, 12 were positive for both *mec*A and *mec*C. The MRSA isolates showed multidrug resistance. The findings showed high prevalence of *mec*C-positive LA-MRSA in Malaysia and highlight the public health risks to people that may come in contact with the carrier animals or those who may consume unpasteurized milk products from these animals.

## 1. Introduction

*Staphylococcus aureus* is a normal inhabitant of the skin and mucous membrane of healthy human and animals. However, it can also be opportunistic pathogen and causes multiple infectious diseases in humans and animals [1] and the bacteria can spread through air, contaminated surfaces, animals, or human [2]. It has been widely reported that *S. aureus* is commonly detected in raw milk from both apparently healthy animals and those with clinical mastitis. A recent study from China reported 46.2% (90/195) of raw milk samples taken from dairy cows with mastitis were positive for *S. aureus* [3]. *Staphylococcus aureus* is also known for its multidrug resistance and MRSA is one of the most potent drug resistant bacteria that has been causing nosocomial infections and community associated infections and animal diseases. According to the centers for disease control and prevention (CDC), strains of *S. aureus* that are oxacillin and methicillin resistant are considered resistant to all ß-lactam agents, including cephalosporins and carbapenems. It has been reported that animal MRSA isolates were significantly more resistant to ciprofloxacin, gentamicin, and clindamycin as compared to human MRSA isolates [4]. Recently, a highly divergent *mec*A gene, *mec*C was found in *S. aureus* causing bovine mastitis [5]. This novel LA-MRSA was first reported from cattle in the UK and Denmark where it was reported to cause human and animal infections. In recent years, the *mecC* MRSA/LA-MRSA strains have been reported from a few European countries and from different host species. Molecular characterization and typing showed that some animal MRSA lineages are host specific. In addition to farm animals, MRSA strains of animal origin were also reported to be infectious to humans [6]. Among animal MRSA strains, ST398 is considered as the most notable LA-MRSA strain which was initially found in pigs and was subsequently detected in several companion and food animals and in humans [6]. A study by Bardian et al. reported that ST398 was as a major MRSA clone in milk from cows affected with bovine mastitis in Belgium The same study reported that this strain of MRSA has been spreading to other farm animals, particularly dairy cattle [7]. High prevalence of MRSA has also been reported in dairy cattle from China where 47.6% prevalence was recorded in dairy farms [8]. Relatively lower prevalence of MRSA was reported elsewhere. A study from India reported a 13% prevalence of MRSA in dairy cows [9]. High MRSA prevalence in a dairy farm might be attributed to the imprudent usage of antibiotics and perhaps poor intramammary administration of antibiotics in cows affected by mastitis.

Several studies have reported high MRSA, CC398 strain colonization rates in humans including animal owners, farmers, veterinarians, and abattoir workers. A case-control study conducted in the Netherlands reported that pig or cattle farmers were often carriers of MRSA-ST398 [10]. According to the study reported by Hanselman et al. [11], 7% of veterinarians and 12% of technician attendees at an international veterinary conference were colonized with MRSA ST398. These studies show that transmission of MRSA can occur from human to animal and vice versa and direct exposure to MRSA-positive animals may lead to transmission to humans [12,13]. In most European countries, CC398 remains the most commonly identified type of LA-MRSA. While CC398 strains have been found in livestock across the globe, the epidemiology of livestock-associated *S. aureus* differs in other geographic areas. Several studies in Asian countries such as China, Malaysia, and Thailand have showed that a different strain of MRSA, ST9, appears to be the prominent type of LA-MRSA [14]. A recent study on the epidemiology of *mec*C MRSA in dairy cattle in France revealed that 22% of the dairy cows carried *mec*C-positive MRSA CC130 strains [15]. An earlier study conducted in dairy sheep farms from Italy reported two MRSA isolates, carrying respectively the *mec*A and the *me*cC genes, with an overall MRSA prevalence of 0.7% [16]. However, there are scarcity of data from most Asian countries including Malaysia on the occurrence and prevalence of LA-MRSA in dairy farms, particularly that of MRSA strains harboring the *mec*C gene.

In Malaysia, detection of MRSA in different species of animals had been reported since 1970s. A research done by Aklilu et al. [17] studied MRSA prevalence in veterinary professionals, cats and dogs, and environmental premises in University Veterinary Hospital. The results showed 2/28 (7.1%) staff, 8/100 (8%) of the pets (5/50 (10%) of the dogs and 3/50 (6%) of the cats), and 9/28 (4.5%) of the environmental samples. However, there has been no reported investigation on the occurrence of LA-MRSA, particularly on MRSA strains harboring the novel methicillin resistance gene, *mec*C in dairy cattle in Malaysia and only few studies were reported elsewhere. Therefore, this preliminary study was conducted to investigate the occurrence of LA-MRA in dairy cattle and determine the antibiotic resistance profiles of the MRSA and LA-MRSA (*mec*C-positive) isolates from dairy cattle.

## 2. Materials and Methods

### 2.1. Sample Collection and Preparation

A total of 63 milk and 32 nasal swab samples were randomly collected from dairy cattle farms in Kota Bharu, Kelantan, Malaysia using sterile collection tubes and swabs with transport media. The milk and nasal samples were immediately put in ice box and brought back to bacteriology laboratory at Faculty of Veterinary Medicine, University Malaysia Kelantan and were briefly stored in a chiller at 4 °C and were processed on the same day.

### 2.2. Ethics

This research was reviewed and approved on 27th December 2018 by the animal research ethics committee at the Faculty of Veterinary Medicine, Universiti Malaysia Kelantan.

### 2.3. Isolation and Identification of Staphylococcus Aureus

The swab samples were submerged into 5 ml of tryptone soy broth (TSB) and incubated at 37 °C for 24 h for enrichment. Whereas 3 ml of the milk from each sample were mixed into 7 mL of TSB and incubated as above. The samples were then cultured on blood agar and nutrient agar and incubated at 37 °C for 24 h. After 24 h colony morphology, Gram staining and biochemical tests were used to identify *S. aureus*. Presumptive *S. aureus* were further confirmed by PCR amplification of *S. aureus* specific gene (*nuc*A). The confirmed *S. aureus* colonies were cultured on oxacillin resistant screening agar base (ORSAB) to screen for MRSA. Blue colonies on ORSAB after incubating for 24–48 h were presumptively identified as MRSA and were cultured on nutrient agar to Further confirmation was done by PCR detection of methicillin resistance encoding gene, *mec*A.

### 2.4. Antibiotic Sensitivity Test

Antimicrobial sensitivity test was done using disc diffusion method. Positive MRSA colonies were transferred into normal saline solution to obtain a turbidity equivalent to 0.5 McFarland physiological standard to create the inoculum. The inoculum was then spread onto Mueller Hinton agar (MHA) using sterile swab. Antibiotic discs, amoxicillin (30 µg), oxacillin (1 µg), cefoxitin (30 µg), gentamycin (10 µg), ciprofloxacin (5 µg), enrofloxacin (5 µg), tetracyclines (30 µg), sulphonamides (300 µg), imipenem (10 µg), and chloramphenicol (30 µg) were used. The diameters of zones of inhibition were measured and the antimicrobial susceptibility was determined and interpreted according to the guidelines of clinical laboratory standard institute (CLSI) [18]. Isolates resistant to oxacillin and cefoxitin were presumptively identified as MRSA and further confirmed by PCR amplification of mecA and/or mecC genes.

### 2.5. Polymerase Chain Reaction (PCR)

#### 2.5.1. DNA Extraction

Extraction of the genomic DNA was conducted by using commercial DNA extraction kit, Machery-Nagel DNA, RNA, and Protein Purification Kit (Duren, Germany) following the recommended procedures. The extracted DNA was stored in a freezer at −20 °C until used.

#### 2.5.2. *S. aureus*-Specific Gene Amplification

Confirmation of *S. aureus* was done by amplifying the *nuc*A gene using the primer sequence (Table 1) as described earlier [19]. Two microliters of samples were added to master mix consisted of 20.9 µL nuclease free water, 10 µL 5X buffer, 1.5 µL 50mM MgCl_2_, 0.5 µL 10mM dNTPs, 5 µL of each primer and 0.1 µL Taq DNA polymerase (5 u/µL). The PCR amplification was done using the following protocols, initial denaturation at 94 °C for 5 min, 35 amplification cycles consisting of denaturation at 94 °C for 30 s, annealing at 62 °C for 45 s and extension at 72 °C for 45 s, followed by final extension at 72 °C for 10 min.

#### 2.5.3. Amplification of Methicillin-Resistance Encoding Gene (mecA)

Methicillin resistant *S. aureus* specific gene, *mec*A (Table 1) was amplified to confirm MRSA isolates according as described previously [20]. Two microliters of sample was added to 48µl of master mix consisted of 26.5 µL nuclease free water, 10 µL 5X buffer, 2 µL 50 mM MgCl_2_, 1 µL 10 mM dNTPs, 3.75 µL of both forward and reverse primers and 1 µL Taq DNA polymerase (5 u/µL). PCR amplification was done using the following protocol, pre-denaturation 1 min, denaturation at 94 °C for 1 min, annealing at 60 °C for 1 min, extension at 72 °C for 3 min and final extension at 72 °C for 5 min. Amplification products yielding 533 bp were considered as positive.

#### 2.5.4. Amplification of LA-MRSA-Specific Gene (mecC)

Livestock associated Methicillin resistant *S. aureus* harboring *mec*C gene were identified by conducting PCR on all phenotypically identified MRSA isolates with positive growth on MRSA selective agar, ORSAB. Specific primers for *mec*C genes (Table 1) as described earlier [21] were used to identify *mec*C positive LA-MRSA isolates. Two microliters of sample were added to 48 µL of master mix containing 26.5 µL nuclease free water, 10 µL 5X buffer, 2 µL 50 mM MgCl2, 1 µL 10 mM dNTPs, 3.75 µL for both 10 µM *mec*C R and *mec*C F and 1 µL Taq DNA polymerase (5 u/µL). The PCR protocol was set as pre-denaturation at 95 °C for 2 min, 30 cycles of amplification with denaturation at 95 °C for 45 s, annealing at 55 °C for 1 min, extension at 72 °C for 2 min and final extension at 72 °C for 5 min. The PCR products were analyzed by gel electrophoresis using 1.2% agarose and gel imaging was done using Gel Doc^TM^ EZ Imager (Bio-Rad, Hercules, CA, USA). The expected amplification product of 304 bp signifies a positive detection of *mec*C gene.

## 3. Results

### 3.1. Isolationand Identification of S. aureus

**Figure 1:**
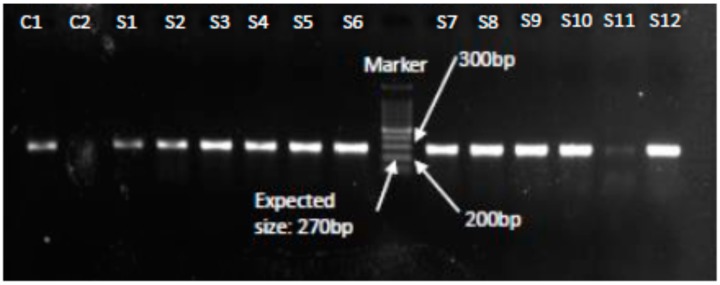
PCR confirmation of S. *aureus* isolates showing amplification of the *nuc*A gene at 270 bp. Lanes C1 and C2 represent positive and negative controls respectively, Lanes S1–S12 show *nuc*A gene positive (270 bp) results confirming *S. aureus*.

The results showed that 44.4% (28/63) of the milk samples and 50% (16/32) of the nasal swabs were positive for *S. aureus* as confirmed by PCR. Overall, the detection rate of *S. aureus* was 46.3% (44/95) (Table 2 and Figure 1).

### 3.2. Identification and Confirmation of MRSA

**Figure 2:**
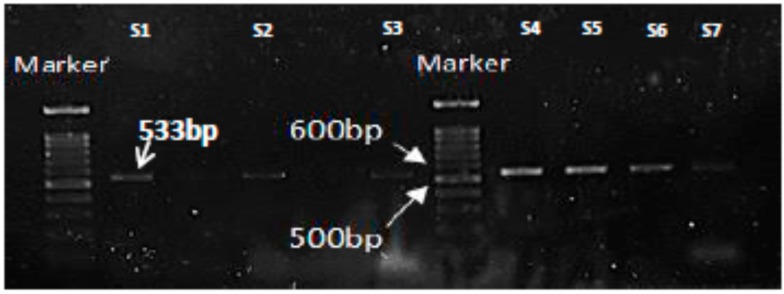
PCR results for amplification of MRSA-specific gene, *mec*A showing the expected product at 533bp. Lane S1 is positive control and Lanes S2-S7 are representative *mec*A positive isolates.

Polymerase chain reaction analysis of 28 *S. aureus* isolates from milk samples and 16 *S. aureus* isolates from nasal swabs samples showed that 46.23% (13/28) and 25% (4/16) isolates were positive for *mec*A gene respectively (Table 3, Figure 2).

### 3.3. Detection of mecC Positive LA-MRSA Isolates

**Figure 3:**
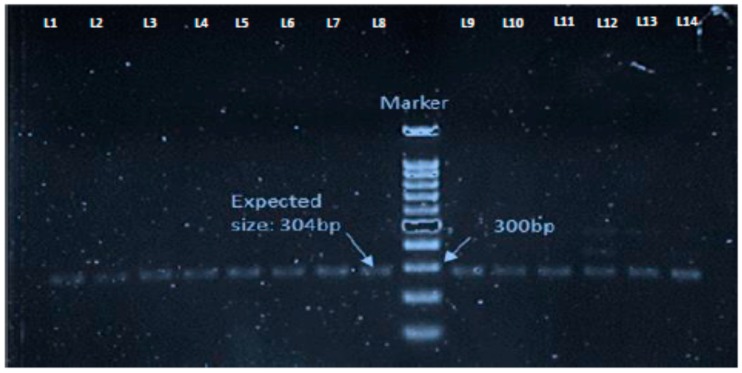
PCR results showing *mec*C gene positive livestock associated Methicillin resistant *Staphylococcus aureus* (LA-MRSA) isolates with the amplification of the expected size, 304 bp. Lanes L1–L14 represent the samples.

Presence of the *mec*C gene is used for confirmation of the novel MRSA strains harboring this specific methicillin resistance encoding gene [22]. Among all positive isolates on ORSAB agar, 15 isolates were positive for *mec*C gene. Out of the 15 *mec*C positive isolates, 12 were also positive for *mec*A gene (Table 4 and Figure 3).

### 3.4. Antibiotic Resistance Profile of MRSA Isolates

**Figure 4:**
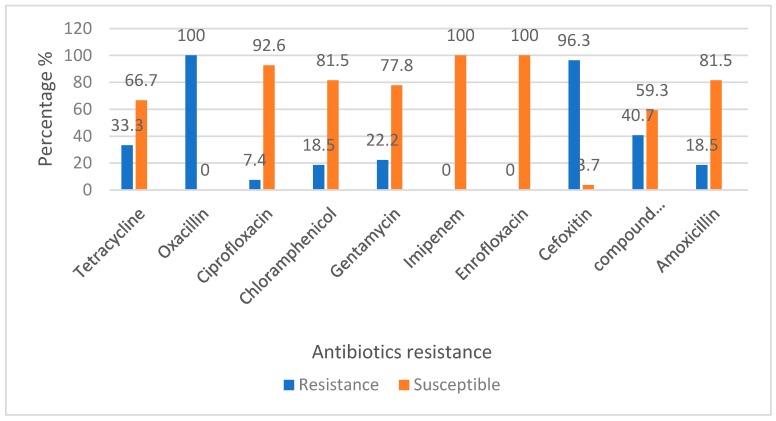
Percentage of antibiotic sensitivity of MRSA from dairy cattle in Kelantan.

Out of 26 MRSA isolates, almost all were resistant towards oxacillin (OX 1, 100%) and cefoxitin (FOX 30, 96.3%). However, all the MRSA isolates were susceptible to Imipenem (IPM 10, 100%) and Enrofloxacin (ENR 5, 100%). Out of all the MRSA isolates, 25 (96.15%) were resistant to at least one type of antibiotics showing multidrug resistance (Figure 4).

## 4. Discussions

In this study, out of the total 95 samples, 44 samples (46.3%) were positive for S. *aureus and* 17 (38.6%) of these were confirmed to be MRSA. Likewise a recent study of *S. aureus* isolates obtained from bovine mastitic milk samples in Bangladesh reported a high prevalence of 20% (29/145) MRSA identified by the presence of the *mecA* gene [23]. In contrast, a more recent study from China reported 15.52% of the 219 bovine mastitis *S. aureus* isolates were confirmed as MRSA by cefoxitin disc diffusion test, oxacillin microdilution test, and *mec*A detection [24]. A relatively lower prevalence rates of MRSA were also reported from other studies conducted in in different countries. Including US and China [25,26,27]. A study from Italy reported that 3.8% (40/484) *S. aureus* isolates from milk and milk products were MRSA [28], whereas another study from the same country reported that out of 169 *S. aureus* strains isolated from Italian dairy cows, 12 (7.1%) and 157 (92.9%) were MRSA and methicillin susceptible *S. aureus* (MSSA) respectively [29].

Different prevalence rates of bovine MRSA has been reported in many countries. Huber et al. [30] reported a low prevalence of MRSA in bovine milk (2 out of 142 *S. aureus* isolates) in Switzerland. In Germany 16.7% of prevalence rate was detected [31] and 0.4% in Hungary [32]. In a recent study by Paterson et al. [33], 7 MRSA isolates were detected in 1500 bulk milk tank samples tested in UK. Whereas 4.4% of the analyzed bulk milk samples in Germany were positive for MRSA [34]. Some of the Asian countries have also reported the occurrence of bovine MRSA. Pu et al. [8] reported 47.6% prevalence in China, while others reported 6.3% in Korea [13], 13.1% in India [9] and 1.5% in Japan [35]. These reports imply that Asian countries have relatively higher prevalence rates of bovine MRSA as compared to European countries and Malaysia is among the Asian countries that has reported high prevalence of bovine MRSA and these findings might be attributed to possible imprudent usage of antibiotics.

High percentage of MRSA isolated from dairy cattle in this study might be due to the fact that antibiotics are extensively used to control and prevent bacterial infections such as bovine mastitis. The indiscriminate use of antibiotics may lead to the emergence of multidrug-resistant bacterial strains and increases the risk of presence of residues of these drugs in milk [36]. Detection of high percentage of MRSA from dairy cattle also shows that there is high risk of potential zoonotic transmission especially to the farmers, veterinarians handling the livestock and to the public who may consume the dairy products that are not processed properly. This is because MRSA infected cattle can act as a reservoir of MRSA and may transmit the bacteria to other animals and humans [37,38].

In this study, out of 95 samples, a total of 15 (57.69%) livestock associated methicillin resistant *Staphylococcus aureus* (LA-MRSA) harboring the novel *mec* gene, *me*cC were detected by PCR. Out of the 15 positive isolates, 11 were positive for both *mec*A and *mec*C genes. This shows that LA-MRSA possessing *mec*C gene is not only present in European countries but also in Asian, particularly in Malaysia. To the best of our knowledge, the current finding is the first report on LA-MRSA (*mec*C positive) in dairy cattle in Malaysia and is among the few reports of *mec*C positive LA-MRSA outside Europe. This high percentage of mecC positive LA-MRSA in dairy cattle shows that there is high risk for zoonotic transmission of this pathogen to veterinarians or farmers because of its capacity to colonize a wide range of hosts [5]. Studies have reported that MRSA colonization in cattle may be an occupational risk to veterinarians, farmers, milkers, and people working at slaughterhouses [5,32]. It has also been reported that the transmission of animal MRSA to veterinary personnel can occur and such transmission commonly occurs in personnel working with large animals [11,39,40].

In this study, different groups of antibiotics were tested, and the results show that all the MRSA isolates were resistant to oxacillin, but are susceptible to imipenem and enrofloxacin. Moreover, all the isolates were resistant towards at least one type of antibiotics, showing multi-drug resistance characteristics of the MRSA isolates. Among others, the possible explanation for the MRSA isolates being resistant to penicillin and other similar antibiotics can be due to the fact that these groups of antibiotics are commonly used by farmers and veterinarians in treating dairy cattle especially for disease such as mastitis. To prevent the incidence of antibiotic resistance in dairy cattle from rising, surveillance for early identification of novel antibiotic resistant clones of *S. aureus* is recommended [41]. It is also important to improve biosecurity and implement good animal husbandry practices in dairy farms to prevent the spread of MRSA and other antimicrobial resistant pathogenic bacteria. Due to the zoonotic potential of LA-MRSA, veterinarians and farm workers are advised to adhere to safety procedures including usage of personal protective equipment whenever handling the animals.

In conclusion, the findings from the current study are preliminary and more studies needs to be conducted for further identification of the *mec*C positive isolates through additional molecular characterization and typing techniques. Nevertheless, the isolation of *mec*C positive LA-MRSA from dairy cattle in this study is the first such report on the detection of this MRSA strain in Malaysia and is expected to serve as a preliminary data to initiate comprehensive and large-scale research. The information generated from this study is important to understand the presence of this bacteria in dairy cattle and determine the public health risks it may pose, mainly to the animal owners, people who may come in contact with carrier animals and those who may consume unpasteurized dairy products. Moreover, the data from this research can also be used to educate the public on the potential public threat posed by LA-MRSA from dairy cattle and milk products from these animals.

## Figures and Tables

**Table 1:** Primers used in this study.

Isolate	Target Gene	Primer Sequence	Amplification Products (bp)	Reference
*S.aueus*	*nuc*A	F 5′-GCGATTGATGGTGATACGGTT-3′ R5′-AGCCAAGCCTTGACGAACTAAAGC-3′	270	[19]
MRSA	*mec*A	F 5′-CCTAGTAAAGCTCCGGAA-3′ R 5′-CTAGTC-CATTCGGTCCA-3′	533	[20]
LA-MRSA	*mec*C	F 5′TGTTGTAGCAATGTTCACAC-3′ R 5′CAAGCACTTAATATCAACGC-3′	304	[21]

**Table 2:** Percentage of positive *Staphylococcus aureus* isolated from different samples of dairy cattle.

Source	Samples	Positive for *nuc*A	Percentage of *S. aureus (%)*
Milk	63	28	44.4
Nasal swabs	32	16	50.0
Total	95	44	46.3

**Table 3:** Percentage of positive Methicillin resistant *Staphylococcus aureus* (MRSA) isolates from different samples of dairy cattle.

Source	Samples	Positive ORSAB	Positive for *mec*A	Percentage of MRSA (%)
Milk	63	22	13	20.63
Nasal swabs	32	4	4	12.50
Total	95	26	17	17.89

**Table 4:** Percentage of positive Livestock associated Methicillin resistant *S. aureus* isolates.

Source	Samples	Positive ORSAB	Positive *mec*C Gene	Percentage LA-MRSA (%)	Positive for both *mec*A and *mec*C Genes
Milk	63	22	11	17.46	8
Nasal swabs	32	4	4	12.50	4
Total	95	26	15	15.79	12
